# Idiopathic pneumatosis cystoides intestinalis of the small bowel with perforation and congenital duodenal membranous stenosis and malrotation of intestine. A case report

**DOI:** 10.3389/fsurg.2026.1921559

**Published:** 2026-09-03

**Authors:** Linsheng Zhu, Tingchong Zhang

**Affiliations:** 1 Department of Pediatric Surgery, Shunyi Maternal and Children's Hospital of Beijing Children's Hospital, Beijing, China; 2 Department of Genenal Pediatric Surgery, Beijing Children's Hospital, Capital Medical University, Beijing, China

**Keywords:** case report, children, congenital duodenal membranous stenosis, malrotation of intestine, pneumatosis cystoides intestinalis

## Abstract

**Introduction:**

Pneumatosis cystoides intestinalis (PCI) in children is a disorder characterized by gas-filled cystic lesions located in the submucosa and subserosa of any segment of the gastrointestinal tract, with pneumoperitoneum occurring in partial cases. This disease is well recognized as an early imaging sign of necrotizing enterocolitis secondary to intestinal ischemia in preterm neonates, yet PCI is rare in older children. Unlike pneumatosis intestinalis associated with life-threatening conditions such as neonatal necrotizing enterocolitis and mesenteric ischemia, PCI is generally regarded as a benign disorder. Conservative non-surgical management is indicated for PCI patients without critical life-threatening complications.

**Case presentation:**

We present the case of a 11-year-old female patient presenting with recurrent vomiting and abdominal distension for 9 years. She was diagnosed with congenital congenital duodenal membranous stenosis and underwent laparoscopic surgery at our hospital. Intraoperative findings included congenital duodenal membranous stenosis, extensive pneumatosis involving the lesser curvature of the stomach and ileal wall with alveolar air cysts of variable morphology, as well as intestinal contents extravasated secondary to intestinal perforation. Congenital malrotation of the intestine was also identified. The patient received laparoscopic resection of duodenal web combined with duodenal longitudinal incision and transverse plasty, plus reduction of intestinal malrotation; no specific intervention was performed for the intestinal gas cysts. Postoperative diagnoses: congenital duodenal membranous stenosis, pneumatosis cystoides intestinalis, congenital intestinal malrotation. The patient was followed up for 29 months postoperatively. At present, she has no special discomforts such as abdominal pain or vomiting, with markedly relieved abdominal distension, and remains under regular follow-up.

**Discussion:**

We discussed whether the lesion distribution of pediatric PCI is correlated with elevated intraluminal gastrointestinal pressure. Pediatric PCI carries a risk of intestinal perforation; non-surgical management can be adopted after exclusion of life-threatening critical conditions.

**Conclusion:**

The diagnosis and management of pediatric pci are clinically challenging due to the potential risk of perforation, while most concurrent pneumoperitoneum is benign. its pathogenesis may be associated with partial gastrointestinal obstruction and increased intraluminal pressure. regular follow-up and reexamination are recommended for children without life-threatening manifestations, and non-surgical treatment is preferred in the absence of acute abdomen.

## 1 Introduction

Pneumatosis cystoides intestinalis (PCI) is a disease characterized by gas-containing cystic blebs located in the submucosa and subserosa of the intestinal wall (1). Although PCI is well recognized as an early imaging marker of intestinal ischemia-induced necrotizing enterocolitis in preterm neonates, it remains rare in older children (2). Unlike intestinal pneumatosis secondary to life-threatening conditions including neonatal necrotizing enterocolitis and mesenteric ischemia, PCI is generally considered a benign disorder. Only a small subset of PCI cases are primary or idiopathic, while the majority are secondary to a variety of underlying disorders. Clinical manifestations are non-specific, and some patients may even be entirely asymptomatic. Conservative non-surgical management is recommended for PCI patients without obvious life-threatening complications (3). The incidence is higher in males than females, and the colon is more frequently involved than the small intestine (4). PCI can be associated with numerous underlying disorders; in pediatric populations, relevant predisposing conditions include chemotherapy, immunosuppressive therapy, long-term high-dose corticosteroid administration, inflammatory bowel disease, congenital heart disease, and intestinal motility disorders (1, 5, 6). Approximately 15% of all PCI cases are idiopathic. Affected patients may be asymptomatic or present with vague non-specific complaints. Complications such as intestinal obstruction and perforation occur in 3%–16% of PCI cases (3). Abdominal radiography yields diagnostic findings in roughly two-thirds of patients, whereas computed tomography (CT) demonstrates superior sensitivity compared with radiography and ultrasonography (7). Herein, we present the case of an 11-year-old child with a 9-year history of recurrent vomiting and abdominal distension, who underwent surgical intervention for radiologically confirmed congenital duodenal membranous stenosis and was intraoperatively diagnosed with pneumatosis cystoides intestinalis.

## 2 Case report

**Figure 1:**
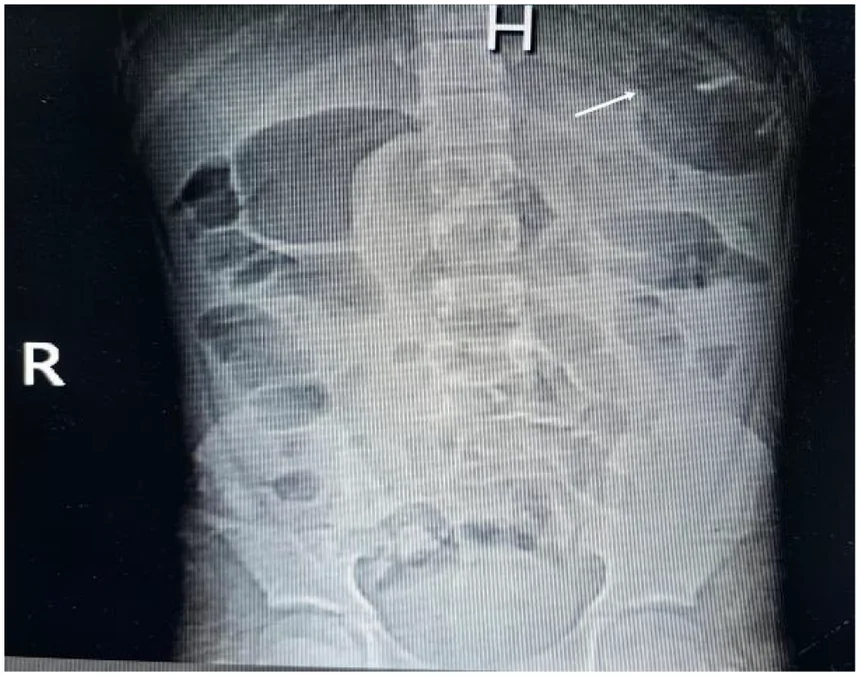
(Left) free subdiaphragmatic gas on the right side.

**Figure 2:**
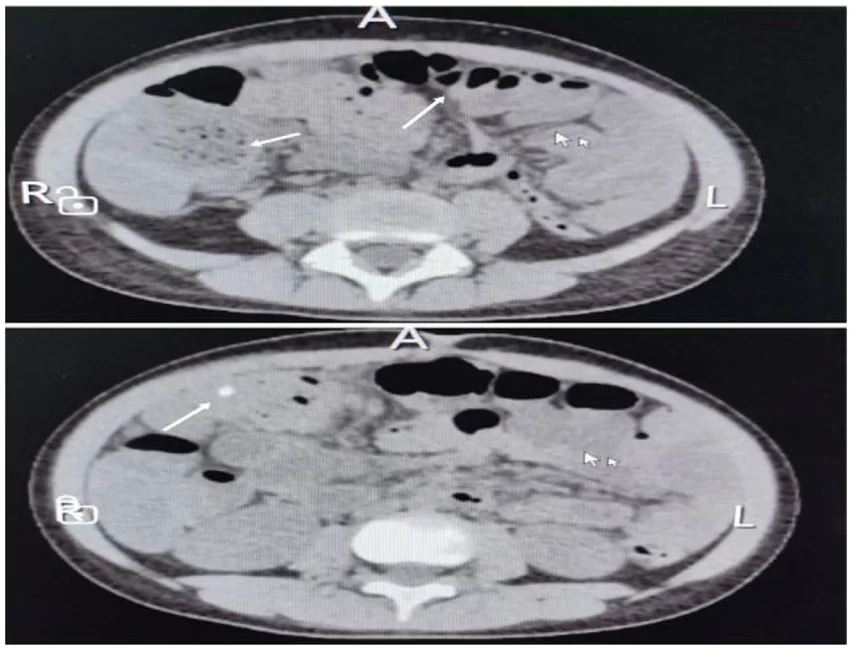
(Right) extensive gas accumulation within the gastrointestinal tract and a round-like high-density shadow.

**Figure 3:**
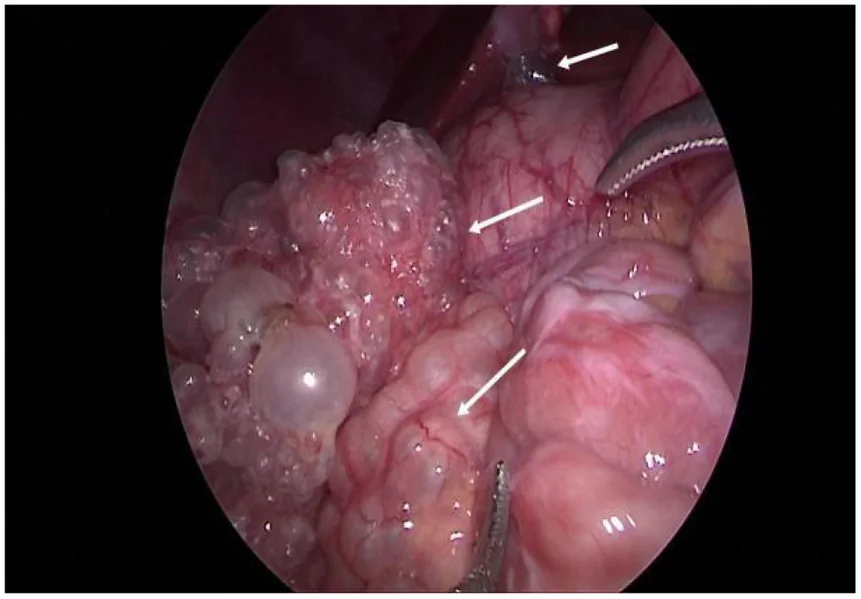
(Left) intraoperative view showing gas cysts on the lesser gastric curvature and ileum.

**Figure 4:**
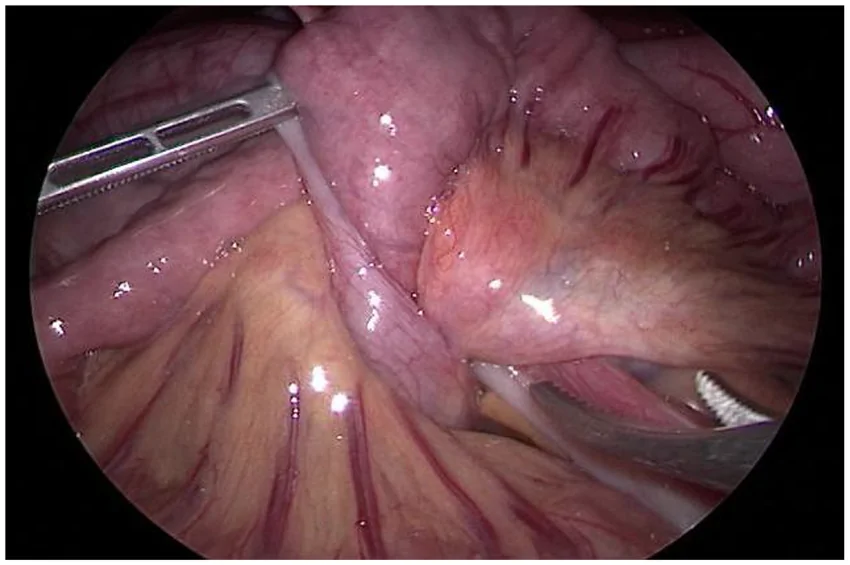
(Right) intraoperative appearance of intestinal malrotation.

**Figure 5:**
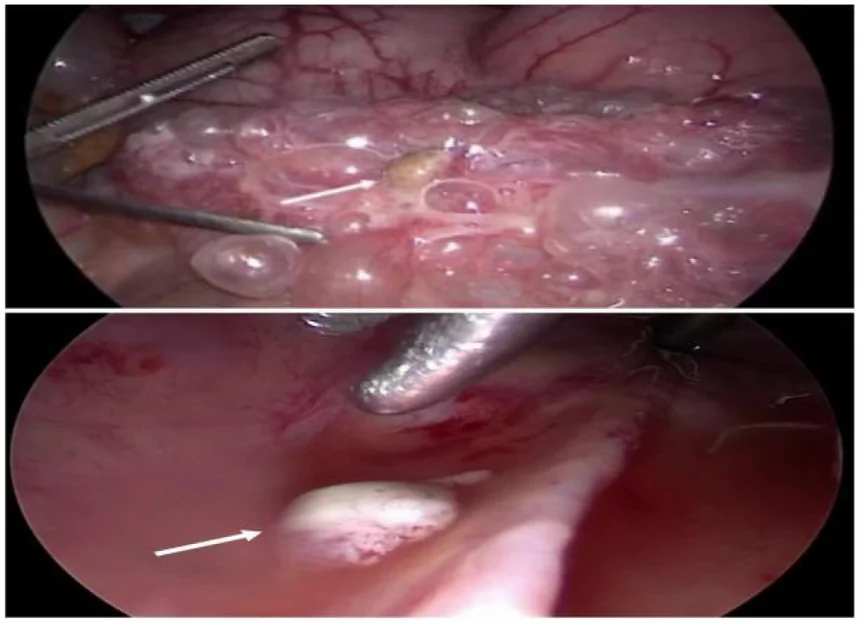
(Left) intraoperative view showing intestinal contents within the gas cysts and a free-floating foreign body.

**Figure 6:**
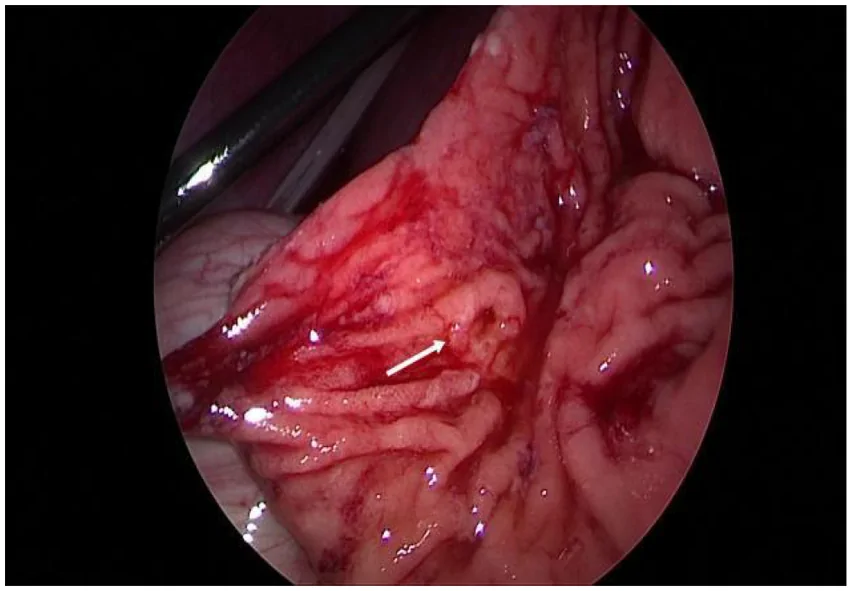
(Right) longitudinal incision of the anterior duodenal wall revealing the orifice of congenital duodenal membranous stenosis.

We report the case of an 11-year-old female patient with a 9-year history of vomiting and abdominal distension. She was diagnosed with congenital duodenal membranous stenosis via abdominal ultrasonography and upper gastrointestinal contrast radiography at Beijing Children's Hospital, Capital Medical University, but did not receive surgical treatment at that time. The patient underwent corrective surgery for congenital heart disease at Beijing Anzhen Hospital more than 9 years ago, strabismus correction surgery at Beijing Children's Hospital over 2 years ago, and laparoscopic high ligation of bilateral hernial sacs at Qingdao Women and Children's Hospital more than 1 year prior. During the hernia operation, follicular dilatation of small intestinal lymphatic vessels was incidentally detected without any intraoperative intervention. Recently, the patient suffered progressively aggravated abdominal distension, abdominal pain and vomiting. Physical examination revealed marked abdominal distension predominantly in the mid-upper abdomen with hyperresonance on percussion. Abdominal gastrointestinal ultrasonography confirmed congenital duodenal membranous stenosis. Plain radiography and CT (Figure 1, Figure 2) revealed free subdiaphragmatic gas on the right side and extensive gas accumulation within the gastrointestinal tract and a round-like high-density shadow. The patient underwent laparoscopic surgery at our institution. Intraoperative exploration demonstrated extensive pneumatosis involving the lesser gastric curvature and ileal wall, with alveolar, beaded gas cysts as well as incompletely clustered gas vesicles (Figure 3). Concurrent congenital intestinal malrotation was also identified (Figure 4). During ileal inspection, intestinal luminal-like material and a free-floating foreign body was observed within the intramural gas cysts (Figure 5). Combined with the presence of subdiaphragmatic free gas, a remote history of intestinal perforation was suspected. Laparoscopic resection of the duodenal web, duodenal longitudinal incision with transverse plasty, and reduction of intestinal malrotation were performed (Figure 6), and no targeted intervention was administered for the intestinal gas cysts. Postoperative diagnoses: congenital duodenal membranous stenosis, pneumatosis cystoides intestinalis, congenital intestinal malrotation. The patient achieved uneventful postoperative recovery. At the 29-month postoperative follow-up, she remained free of abdominal pain, vomiting and other discomforts, with substantially alleviated abdominal distension, and is maintained on regular outpatient surveillance.

## 3 Discussion

In 1730, French pathologist Du Vernoi first described the presence of gas accumulation in the intestinal wall during autopsy (8), and the disorder he documented is now known as pneumatosis cystoides intestinalis (PCI). PCI is characterized by gas-filled cysts located in the intestinal wall, predominantly within the subserosa or submucosa (9), and may arise at any segment of the gastrointestinal tract. Patients with PCI may be asymptomatic or present with non-specific symptoms such as abdominal pain, diarrhea, abdominal distension, constipation, bloody stool,loss of appetite, and weight loss (10).

In terms of gender distribution, the incidence is higher in males than females. Previous studies have reported male-to-female ratios of 3.5:1 and 3:1, while a population-based Chinese study reported a ratio of 2.4:1 (3).

PCI is a well-defined radiographic sign of necrotizing enterocolitis in neonates, primarily observed in preterm infants. In contrast, the pathogenic mechanisms of PCI in children remain poorly elucidated, and there is no consensus regarding its clinical significance and standardized diagnostic and therapeutic regimens (11, 12). Although multiple hypotheses have been proposed to explain its pathogenesis, the exact pathophysiology of PCI has not been fully clarified to date, with three predominant theories: the pulmonary origin theory, mechanical theory, and bacterial theory.

The pulmonary origin theory postulates that gas originates from the thoracic cavity; following alveolar rupture, gas dissects along vascular tracts into the retroperitoneal space and mesenteric root (7). However, intraoperative observation of the present case revealed that gas cysts were almost exclusively distributed on the antimesenteric side of the bowel, with no abnormalities identified at the mesenteric root.

The mechanical theory proposes that gas derives from the intestinal lumen, generated by the combined effects of elevated intraluminal gastrointestinal pressure and increased mucosal permeability. In this case, gas cysts were mainly localized to the lesser curvature of the stomach and ileum, which may be associated with impaired intestinal transit and elevated intraluminal pressure secondary to membranous duodenal stenosis and malrotation.

The bacterial theory holds that gas produced by gas-forming bacteria penetrates the intestinal wall through damaged mucosa. In immunocompromised pediatric patients, fragile intestinal wall architecture may facilitate gas migration into the peritoneal cavity (13).

The true incidence of PCI remains undetermined, as most cases are detected incidentally, including the present case. Nearly all cases are identified fortuitously via plain abdominal radiography or computed tomography (CT), Radiological imaging plays an essential part for thediagnosis of PCI. Cysts usually appear as honeycomb signs or grape-shaped clusters along the intestinal wall under radiological imaging (14).

Whereas colonoscopy can detect PCI in adult patients. Colonoscopy also is the main diagnostic method for colonic PCI. Under endoscopy, PCI can be presented as vacuolated, botryoid or beaded, linear or cobblestone pattern, and irregular. Vacuolated lesions tend to be primary and should be differentiated from polyps, botryoid or beaded lesions should be differentiated from intestinal tuberculosis, while irregular lesions should be differentiated from Crohn's disease and tumors (4).

CT represents the gold standard for the radiological diagnosis of PCI and may also identify underlying or concomitant diseases in a subset of patients. The pathognomonic radiographic feature of PCI is cystic or bubbly gas deposition within the intestinal wall. In comparison, pneumatosis intestinalis secondary to mesenteric ischemia typically demonstrates linear gas tracking along the bowel wall. Rupture of subserosal cysts may lead to pneumoperitoneum, which is generally termed benign pneumoperitoneum in asymptomatic children. Pneumoperitoneum complicates small intestinal PCI at a rate of 15%, compared with 2% for colonic PCI. Other associated complications include intestinal obstruction and bowel perforation (3). In benign PCI lesions, emphysema is transient; gas gradually resolves from the intestinal wall and can be managed conservatively. Incidental asymptomatic PCI requires no aggressive intervention and is likely to regress spontaneously (15). The appropriate therapy is related to the underlying cause of PCI. The majority of patients without pronounced symptoms were cured without any treatments. If the symptoms are pronounced, a conservative approach to treatment is allowed, such as gastrointestinal decompression, intestinal ‘rest’', parenteral nutrition, and ﬂuid and electrolyte supplementation. However, in contrast to the case reports, we found the efficiency of treatment by antibiotics was only 26.3%. The most effcient treatment was therapeutic alliance (16). Patients with moderate to severe symptoms may require endoscopy or laparotomy if still symptomatic despite medical treatment or if they show clinical signs of deterioration or medical instability (17).

Intraperitoneal exploration in this case suggested a remote history of bowel perforation. Given the self-limiting nature of symptoms and absence of life-threatening manifestations, a non-operative management strategy was adopted. The patient has been followed up for 29 months to date, with no recurrent abdominal pain, vomiting or other adverse symptoms, and marked improvement in abdominal distension.

## 4 Conclusions

Diagnosis and management of pneumatosis cystoides intestinalis (PCI) in children pose clinical challenges. Bowel perforation may occur in such patients, and pneumoperitoneum in most cases is benign. The pathogenesis is likely associated with partial gastrointestinal obstruction and elevated intraluminal pressure. For pediatric patients without overt life-threatening conditions, regular follow-up and reexamination are recommended, and non-operative treatment can be adopted in the absence of acute abdomen.

## Data Availability

The original contributions presented in the study are included in the article/Supplementary Material, further inquiries can be directed to the corresponding author.

## References

[1] Galea J Burnand KM Dawson FL Sinha CK Rex D Okoye BO. Pneumoperitoneum in the setting of pneumatosis Intestinalis in children: is surgery always indicated? Eur. J. Pediatr. Surg. (2017) 27:12–5. 10.1055/s-0036-1587335 27595440

[2] Tang ML Williams LW. Pneumatosis intestinale in children with primary combined immunodeficiency. J. Pediatr. (1998) 132:546–9. 10.1016/S0022-3476(98)70040-X 9544921

[3] Strahm R Pratsinis A Jochum AK De Lorenzi D Idiopathic pneumatosis cystoides intestinalis of the small bowel with pneumoperitoneum and consecutive small bowel mesenteric torsion. A case report. Int J Surg Case Rep. 2024;115:109220. 10.1016/j.ijscr.2024.109220 38194864 PMC10819719

[4] Wang YJ Wang YM Zheng YM Jiang HQ Zhang J. Pneumatosis cystoides intestinalis: six case reports and a review of the literature. BMC Gastroenterol. (2018) 18:100. 10.1186/s12876-018-0794-y 29954324 PMC6022295

[5] Kurbegov AC Sondheimer JM. Pneumatosis intestinalis in non-neonatal pediatric patients. Pediatrics. (2001) 108:402–6. 10.1542/peds.108.2.402 11483806

[6] Schuster B Mayr J. Acute pneumatosis cystoides intestinalis with atrophic desmosis of the colon in a child. BMJ Case Rep. (2017,) 2017:bcr-2017–219310. 10.1136/bcr-2017-219310 PMC5534828 28630222

[7] Peter StSD Abbas MA Kelly KA. The spectrum of pneumatosis intestinalis. Arch Surg. (2003) 138 (1):68–75. 10.1001/archsurg.138.1.68 12511155

[8] Du Vernoy JG. Aer intestinorum tam sub extima quam intima tunica inclusus: observationes anatomicae: comment, acad. Acient Imp Petropol. (1730) 5:213–5.

[9] Heng Y Schuffler MD Haggitt RC Rohrmann CA. Pneumatosis intestinalis: a review. Am J Gastroenterol (1995) 90:1747–58. 7572888

[10] Valle JW Faivre S Hubner RA Grande E Raymond E. Practical management of sunitinib toxicities in the treatment of pancreatic neuroendocrine tumors. Cancer Treat Rev. (2014) 40(10):1230–8. 10.1016/j.ctrv.2014.09.001 25283354

[11] Nellihela L Mutalib M Thompson D Jochen K Upadhyaya M. Management of pneumatosis intestinalis in children over the age of 6 months: a conservative approach. Arch. Dis. Child. (2018) 103:352–5. 10.1136/archdischild-2017-313201 28988213

[12] Abramov A Luks VL De Bie F Hwang R Allukian M,3 Nace GJ. Pneumatosis intestinalis in children beyond the neonatal period: is it always benign? Pediatr. Surg Int. (2022) 38:399–407. 10.1007/s00383-021-05048-0 34837497

[13] Ling F Guo D Zhu L. Pneumatosis cystoides intestinalis: a case report and literature review. BMC Gastroenterol. (2019) 19:176. 10.1186/s12876-019-1087-9 31694581 PMC6836417

[14] Ogul H Pirimoglu B Kisaoglu A Karaca L Havan N Ozogul B. Pneumatosis cystoides intestinalis: an unusual cause of intestinal ischemia and pneumop Int Surg. (2015) 100(2):221–4. 10.9738/INTSURG-D-13-00238.1 25692421 PMC4337433

[15] Pickhardt PJ Kim DH Taylor AJ. Asymptomatic pneumatosis at CT colonography: a benign self-limited imaging finding distinct from perforation. AJR Am J Roentgenol. (2008) 190:W112–7. 10.2214/AJR.07.2843 18212192

[16] Wu LL Yang YS Dou Y Liu QS. A systematic analysis of pneumatosis cystoids intestinalis. World J Gastroenterol. (2013) 19(30):4973–8. 10.3748/wjg.v19.i30.4973 IF: 7.7 Q1 23946603 PMC3740428

[17] Habeb B Khair S Fowler M. A rare case of postoperative pneumatosis Intestinalis complicated by sigmoid colon perforation requiring immediate surgical intervention. Cureus. (2025) 17(2):e78480. 10.7759/cureus.78480 40051934 PMC11884214

